# The molecular evolutionary dynamics of oxidative phosphorylation (OXPHOS) genes in Hymenoptera

**DOI:** 10.1186/s12862-017-1111-z

**Published:** 2017-12-28

**Authors:** Yiyuan Li, Rui Zhang, Shanlin Liu, Alexander Donath, Ralph S. Peters, Jessica Ware, Bernhard Misof, Oliver Niehuis, Michael E. Pfrender, Xin Zhou

**Affiliations:** 1 Department of Biological Sciences, University of Notre Dame, Notre Dame, IN USA; 2 Environmental Change Initiative, Notre Dame, IN USA; 3 China National GeneBank, BGI-Shenzhen, Guangdong Province, Shenzhen, China; 4 Centre for GeoGenetics, Natural History Museum of Denmark, University of Copenhagen, Copenhagen, Denmark; 5 Zoologisches Forschungsmuseum Alexander Koenig, Zentrum für Molekulare Biodiversitätsforschung (zmb), Bonn, Germany; 6 Zoologisches Forschungsmuseum Alexander Koenig, Abteilung Arthropoda, Bonn, Germany; 7 Department of Biological Sciences, Rutgers University, Newark, NJ 07102 USA; 8 Center for Molecular Biodiversity Research, Zoological Research Museum Alexander Koenig, Bonn, Germany; 9 Evolutionary Biology and Animal Ecology, Institute of Biology I (Zoology), Albert Ludwig University of Freiburg, Hauptstr. 1, 79104 Freiburg, Germany; 10 Beijing Advanced Innovation Center for Food Nutrition and Human Health, China Agricultural University, Beijing, 100193 China; 11 Department of Entomology, China Agricultural University, Beijing, 100193 China

**Keywords:** Molecular evolution, Positive selection, Mitochondrial-nuclear interaction, Insects

## Abstract

**Background:**

The primary energy-producing pathway in eukaryotic cells, the oxidative phosphorylation (OXPHOS) system, comprises proteins encoded by both mitochondrial and nuclear genes. To maintain the function of the OXPHOS system, the pattern of substitutions in mitochondrial and nuclear genes may not be completely independent. It has been suggested that slightly deleterious substitutions in mitochondrial genes are compensated by substitutions in the interacting nuclear genes due to positive selection. Among the four largest insect orders, Coleoptera (beetles), Hymenoptera (sawflies, wasps, ants, and bees), Diptera (midges, mosquitoes, and flies) and Lepidoptera (moths and butterflies), the mitochondrial genes of Hymenoptera exhibit an exceptionally high amino acid substitution rate while the evolution of nuclear OXPHOS genes is largely unknown. Therefore, Hymenoptera is an excellent model group for testing the hypothesis of positive selection driving the substitution rate of nuclear OXPHOS genes. In this study, we report the evolutionary rate of OXPHOS genes in Hymenoptera and test for evidence of positive selection in nuclear OXPHOS genes of Hymenoptera.

**Results:**

Our analyses revealed that the amino acid substitution rate of mitochondrial and nuclear OXPHOS genes in Hymenoptera is higher than that in other studied insect orders. In contrast, the amino acid substitution rate of non-OXPHOS genes in Hymenoptera is lower than the rate in other insect orders. Overall, we found the *dN*/*dS* ratio of the nuclear OXPHOS genes to be higher in Hymenoptera than in other insect orders. However, nuclear OXPHOS genes with high *dN*/*dS* ratio did not always exhibit a high amino acid substitution rate. Using branch-site and site model tests, we identified various codon sites that evolved under positive selection in nuclear OXPHOS genes.

**Conclusions:**

Our results showed that nuclear OXPHOS genes in Hymenoptera are evolving faster than the genes in other three insect orders. The branch test suggested that while some nuclear OXPHOS genes in Hymenoptera show a signature of positive selection, the pattern is not consistent across all nuclear OXPHOS genes. As only few codon sites were under positive selection, we suggested that positive selection might not be the only factor contributing to the rapid evolution of nuclear OXPHOS genes in Hymenoptera.

**Electronic supplementary material:**

The online version of this article (10.1186/s12862-017-1111-z) contains supplementary material, which is available to authorized users.

## Background

Understanding the patterns and rates of molecular evolution requires consideration of the role of mutation, drift, and selection acting on individual genes. In many cases, the effects of these forces are complicated due to physical and/or functional interaction of the affected genes. A classic system of such interacting genes represents the OXPHOS pathway [1–3]. The OXPHOS pathway is the primary ATP source in eukaryotic cells, generating 70–80% of the ATP demand of cells [4–6]. The OXPHOS pathway comprises five enzyme complexes (complexes I-V), which transport electrons to produce ATP. Complexes I, III, IV, and V are composed of polypeptides encoded by both the mitochondrial and the nuclear genes (Table 1) [2, 7, 8]. Mitochondrial and nuclear OXPHOS genes work together to maintain the ATP production in the cell. Incompatible mitochondrial and nuclear genes can reduce the efficiency of cellular ATP production and contribute to increased oxidative stress, leading to a variety of physiological issues, including developmental abnormalities [3, 9] and reduced hybrid fitness [10, 11]. Interestingly, the mitochondrial genome of animals often show a 5–20 times higher substitution rate than the nuclear genome [12–14] in large part due to fundamental differences between mitochondrial and nuclear genomes in the mode of inheritance, ploidy level, effective population size, and recombination [15–17]. A significant component of the elevated substitution rate in the mitochondrial genome is the lack of recombination, which makes it prone to the accumulation of slightly deleterious mutations [14, 15, 18]. As a result of the rapid rate of molecular evolution and accumulation of deleterious mutations in the mitochondrial genome, it has been suggested that that nuclear OXPHOS genes should be exposed to positive selection for compensatory substitutions that maintain the functional properties of the interacting genes in the OXPHOS system. [2, 6, 19–21].

**Table 1:** Number of OXPHOS genes found in 1KITE data for each complex in this study

Complex	Function	Number of Nuclear OXPHOS Genes	Number of Nuclear OXPHOS Genes Used in This Study	Number of Mitochondrial Genes	Number of Mitochondrial Genes Used in This Study
I	NADH:ubiquinone oxidoreductase	34	16	7	7
II	Succinate dehydrogenase	4	0	0	0
III	Ubiquinol-cytochrome c reductase	9	1	1	1
IV	Cytochrome c oxidase	8	1	3	3
V	ATP synthase	13	5	2	2

A number of studies have examined the patterns of molecular evolution in the OXPHOS system (e.g., [19, 20]). The general pattern that emerges is that species with a high amino acid substitution rates in mitochondrial genes also exhibit a high amino acid substitution rate and an elevated *dN*/*dS* ratio (i.e., the ratio of the number of non-synonymous nucleotide substitutions per non-synonymous site to the number of synonymous nucleotide substitutions per synonymous site) in nuclear OXPHOS genes. This observation of an elevated substitution rate in nuclear OXPHOS genes is consistent with the idea of positive selection driving compensatory mutations in nuclear OXPHOS genes in response to the elevated substitution rate in mitochondrial OXPHOS genes [2, 19, 20]. Beneficial mutations in nuclear OXPHOS genes are likely to be fixed as they maintain the efficiency of the OXPHOS process. Consistent with the positive selection hypothesis, substitutions in nuclear OXPHOS genes are over-represented in residues with critical functional importance, including mitochondrial-nuclear contacting residues [19], and regions in the nuclear genome that are linked to hybrid breakdown [9].

While these previous studies have shown a pattern in the rates of substitution in nuclear OXPHOS genes that is consistent with the positive selection hypothesis, and in some cases have identified sites under positive selection in nuclear OXPHOS genes, it remains unclear to what extent the overall elevated amino acid substitution rate of nuclear OXPHOS genes can be explained by positive selection [19]. The signal of positive selection on nuclear OXPHOS genes is usually weak, with overall *dN*/*dS* ratios less than one [6] and with a small number of sites under positive selection [19]. This weak signature of positive selection fuels an alternative hypothesis that relaxed functional constraint on nuclear OXPHOS genes may lead to an elevated amino acid substitution rate and an elevated *dN*/*dS* ratio in lineages with elevated rates of mitochondrial evolution [6, 22]. Given the functional importance of the OXPHOS system, these genes are likely to be under strong purifying selection, resulting in low *dN*/*dS* ratios. Relaxed selection would partially release genes from this constraint and the resulting elevation in *dN*/*dS* ratios that would be hard to distinguish from an elevation due to positive selection. To dissect the role of positive selection, we focus on an order of insects, Hymenoptera, with notoriously high mitochondrial substitution rates [23]. In this lineage, the effect of positive selection driving nuclear OXPHOS genes should be exaggerated compared to other insects.

The rate of molecular evolution varies substantially across insect orders [24, 25]. Among the four largest insect orders (Coleoptera, Diptera, Lepidoptera and Hymenoptera), the mitochondrial genes of Hymenoptera show a significantly elevated amino acid substitution rate compared to the rate of genes of other insect orders [23], while nuclear-encoded non-OXPHOS genes seem to evolve at similar rates across these orders [26]. Therefore, Hymenoptera is a promising system to understand OXPHOS gene evolution. In addition, while there are a few studies on Hymenoptera showing elevated substitution rates in some nuclear OXPHOS genes, (e.g., genes associated with hybrid incompatibility in a parasitoid wasp, *Nasonia* [27]), the patterns of evolution of OXPHOS genes across a broad range of insect species is lacking.

In this study, we use published transcriptome sequence data [28] to explore the molecular evolutionary patterns of mitochondrial and nuclear OXPHOS genes, focusing on 31 insect species across Hymenoptera and three other mega-diverse insect orders: Coleoptera, Diptera, and Lepidoptera. We test (i) whether the amino acid substitution rate of nuclear OXPHOS genes in Hymenoptera is significantly higher than the rate in the other three insect orders, and (ii) whether the high amino acid substitution rate of nuclear OXPHOS genes is consistent with positive selection exerted by fast evolving interacting mitochondrial OXPHOS genes.

## Methods

### Taxon sampling and sequence acquisition

Our data set comprises transcriptome sequence data from 31 holometabolous insect species (details about sequence data source: Table S2 in Misof et al. [28]), including 6 coleopterans, 8 dipterans, 9 hymenopterans, and 8 lepidopterans (Table 2). Transcriptome sequence data of the pea aphid (*Acyrthosiphon pisum*) was used as an outgroup (Table 2).

**Table 2:** Insect species used in this study

Order	Species
Hemiptera	*Acyrthosiphon pisum*
Hymenoptera	*Acromyrmex echinatior*
Hymenoptera	*Apis mellifera*
Hymenoptera	*Bombus terrestris*
Hymenoptera	*Chrysis viridula*
Hymenoptera	*Cotesia vestalis*
Hymenoptera	*Harpegnathos saltator*
Hymenoptera	*Leptopilina clavipes*
Hymenoptera	*Nasonia vitripennis*
Hymenoptera	*Orussus abietinus*
Coleoptera	*Aleochara curtula*
Coleoptera	*Dendroctonus ponderosae*
Coleoptera	*Gyrinus marinus*
Coleoptera	*Lepicerus* sp.
Coleoptera	*Meloe violaceus*
Coleoptera	*Tribolium castaneum*
Lepidoptera	*Bombyx mori*
Lepidoptera	*Manduca sexta*
Lepidoptera	*Nemophora degeerella*
Lepidoptera	*Parides eurimedes*
Lepidoptera	*Polyommatus icarus*
Lepidoptera	*Triodia sylvina*
Lepidoptera	*Yponomeuta evonymella*
Lepidoptera	*Zygaena fausta*
Diptera	*Anopheles gambiae*
Diptera	*Aedes aegypti*
Diptera	*Bibio marci*
Diptera	*Bombylius major*
Diptera	*Drosophila melanogaster*
Diptera	*Lipara lucens*
Diptera	*Triarthria setipennis*
Diptera	*Trichocera saltator*

Starting with a set of seven reference insect species with sequenced genome (*i. e.*, *Nasonia vitripennis, Apis mellifera, Acromyrmex echinatior, Tribolium castaneum, Bombyx mori, Anopheles gambiae, Drosophila melanogaster*), OrthoDB5 (http://cegg.unige.ch/orthodb5) [29] was used to identify 3284 single-copy ortholog genes. Of these ortholog genes, 23 nuclear OXPHOS genes (Table 1) matched the *Drosophila melanogaster* FlyBase IDs of known OXPHOS genes [7, 8]. A table of FlyBase OXPHOS gene symbols and the corresponding OrthoDB5 ortholog group IDs is provided in Additional file 1: Table S1. A set of 1413 single-copy non-OXPHOS genes were obtained by exploiting the ortholog gene set studied by Misof et al. [28] excluding OXPHOS genes.

Since mitochondrial genes were not included in the gene set compiled by Misof et al. [28], we used a mitochondrial gene annotation pipeline [30] to search for and annotate mitochondrial genes in the 32 transcriptomes (31 holometabolous insect species and 1 outgroup species). The mitochondrial genes of *Acromyrmex echinatior*, *Harpegnathos saltator*, and *Cotesia vestalis* had poor coverage in the assembled transcript libraries. For this reason, we used mitochondrial or nuclear genome assemblies to obtain the mitochondrial gene sequence data. Specifically, we made use of the genome assembly version 2.0 of *Acromyrmex echinatior* [31, 32], the genome assembly version 3.3 of *Harpegnathos saltator* [33], and the assembled mitochondrial genome of *Cotesia vestalis* (GenBank accession number FJ154897.1 [34]). Mitochondrial OXPHOS genes were assigned to one of the OXPHOS enzyme complexes I, III, IV, and V (note that OXPHOS complex II is encoded only by nuclear genes) based on the information from MitoComp2 [8]. In total, we studied 13 mitochondrial OXPHOS genes, 23 nuclear OXPHOS genes, and 1413 nuclear non-OXPHOS genes in this study (Table 1, Additional file 2: Table S2).

### Estimation of the amino acid substitution rate

A custom Perl script (Additional file 3) was used to obtain codon alignments. In particular, nucleotide sequences were first translated into amino acid sequences. The amino acid sequences were then aligned using MUSCLE version 3.8.31 [35] with default parameters. The amino acid sequence alignment was used as a blueprint to infer the corresponding codon alignment. Gblocks version 0.91b [36] was used to remove poorly aligned regions in the resulting nucleotide sequence alignments. Gblocks parameters were set as “-t = c -b4 = 6 -b5 = a -e = −gb1”, meaning that input aligned nucleotide sequences were treated as codon alignment, the minimum length of a block is 6-bp, and gaps are allowed. Codon alignments were translated into the final amino acid alignment with a custom Perl script (Additional file 3) for phylogenetic tree reconstruction and amino acid substitution rate estimation.

To obtain the amino acid substitution rate of each gene, gene trees were reconstructed from the amino acid sequence alignment based on the topology from Misof et al. [28]. The branch length between each ingroup species to the outgroup species (*Acyrthosiphon pisum*) (the distance from tips to root distance of the phylogenetic tree) was used as the amino acid substitution rate of the gene of the ingroup species. Phylogenetic trees were reconstructed using RAxML version 8.2.3 [37] by applying the PROTGAMMAAUTO model option, which automatically selects the best-fitting amino acid substitution model based on the log-likelihood value and approximates across-site rate heterogeneity with a gamma distribution. RAxML “-t” option was used for estimating the branch length based on the given topology. The R commands, “cophenetic” [38] and “read.tree” from R package “ape” version 4.1, were used to retrieve the branch length from a given species.

To test whether Hymenoptera differ in their amino acid substitution rate from that of other insect orders, we concatenated the aligned amino acid sequences of all genes in one of three gene categories (mitochondrial OXPHOS genes, nuclear OXPHOS genes, and nuclear non-OXPHOS genes). Phylogenetic trees were reconstructed based on the aligned concatenated amino acid sequences using RAxML version 8.2.3 [37] with the same setting used to build individual gene trees. The branch length from a given species to the outgroup species (*Acyrthosiphon pisum*) was used as a proxy for the average amino acid substitution rate of the concatenated sequences of the ingroup species. Phylogenetic trees from concatenated sequences were visualized and colored with the R package “ggtree” [39].

The two-cluster test implemented in the program tpcv that is part of the LINTREE package (http://www.personal.psu.edu/nxm2/software.htm) [40] was used to test whether the amino acid substitution rate of Hymenoptera differs from that of other insect orders in the three gene categories. Tpcv was applied on the concatenated sequences and the phylogenetic tree from RAxML to test for statistical differences in amino acid substitution rates of gene categories between Hymenoptera and non-Hymenoptera using the amino acid p-distance and a Z-test (with critical value = 0.05). For the two-cluster test of concatenated mitochondrial OXPHOS sequences, three species were removed (*Aleochara curtula, Lepicerus* sp.*,* and *Nemophora degeerella*) as we failed to identify five mitochondrial genes of these three species from the transcriptome dataset (Additional file 2: Table S2), which limited the number of amino acid sites that could be used in the test.

The pairwise Wilcoxon rank sum test (pairwise.wilcox.test) implemented in R [38] was used to test for differences in the amino acid substitution rates between concatenated amino acid sequences of mitochondrial OXPHOS, nuclear OXPHOS, and nuclear non-OXPHOS gene. The Holm correction [41] was used to adjust the *p*-values for multiple comparisons. Terminal branch length (without the shared ancestoral branch length) was used for Wilcoxon test to avoid potential dependence issue of branch length between species.

### Test for positive selection

- dN/dS ratio test for each gene

We used the branch model in codeml from the PAML package version 4.8 [42] to test whether the *dN*/*dS* ratio of each gene is significantly higher in Hymenoptera than in the other insect orders. An example of the branch test control file is provided as supplementary file (Additional file 3). The tips of the Hymenoptera clade were labeled for the branch test. The null hypothesis of the branch test was that all lineages shared the same *dN*/*dS* ratio. The alternative hypothesis was that Hymenoptera had a different *dN*/*dS* ratio from other lineages. Chi-square test (critical value = 0.05) was used to test whether the alternative hypothesis was significantly better than the null hypothesis based on the maximum likelihood score of each test. If the alternative hypothesis was accepted and if the *dN*/*dS* ratio of Hymenoptera was higher than that of the other lineages, we considered the genes of Hymenoptera having experienced positive selection or to have had relaxed functional constraints. If the alternative model was accepted and if the *dN*/*dS* ratio of Hymenoptera was smaller than that of other lineages, we considered the genes of Hymenoptera to have experienced purifying selection.

b. Codon site-specific positive selection test

To test for positive selection of each codon site in Hymenoptera, we used the branch-site model in the PAML package version 4.8 [43]. The ancestral node of Hymenoptera was labeled as the test group for the positive selection test on mitochondrial, nuclear OXPHOS and nuclear non-OXPHOS genes. An example of the branch-site test control file is provided as supplementary file (Additional file 3). Fisher’s exact test was used to test the differences in the number of sites under positive selection between nuclear OXPHOS and nuclear non-OXPHOS genes.

We also used the mixed effects model of evolution (MEME) test [44] implemented in HyPhy (version 2.220150825beta MP) [45] to identify codon sites under episodic positive selection. As MEME does not require a priori knowledge of lineages under potential positive selection, and still had the same power as the PAML branch-site model [46], we used MEME as an explorative tool to identify sites under the positive selection in mitochondrial and nuclear OXPHOS gene sequences.

c. Amino acid site-specific positive selection test

TreeSAAP version 3.2 [47] was used to test for positive selection at the amino acid level of mitochondrial and nuclear OXPHOS genes. The program measures selection on amino acids using 31 structural and biochemical amino acid properties, such as hydropathy, molecular weight, and polarity. Based on the significance of their property changes, amino acid sites were sorted into eight magnitude categories. Sites in categories ‘6’, ‘7’, and ‘8’ have the most radical amino acid changes and were considered to have been under positive selection. Sites under positive selection found on the ancestral node of Hymenoptera were used as the Hymenoptera-specific sites under positive selection. IMPACT_S version 1.0.0 [48] was used to summarize the location of amino acid sites under positive selection from the TreeSAAP results.

## Results

### The amino acid substitution rate among insect orders

**Fig. 1:**
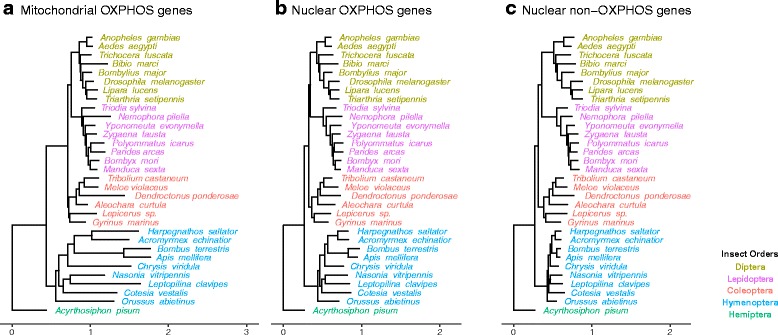
Phylogenetic trees based on concatenated amino acid alignments of (**a**) Mitochondrial OXPHOS genes, (**b**) Nuclear OXPHOS genes, and (**c**) Nuclear non-OXPHOS genes. Species from each order are labelled in different colors (Hemiptera: green, Hymenoptera: blue, Coleoptera: red, Lepidoptera: pink, Diptera: brown). Branch lengths are scaled to the average number of amino acid substitutions per site. Phylogenetic trees were reconstructed based on the topology from Misof et al. [28] with RAxML version 8.2.3

**Fig. 2:**
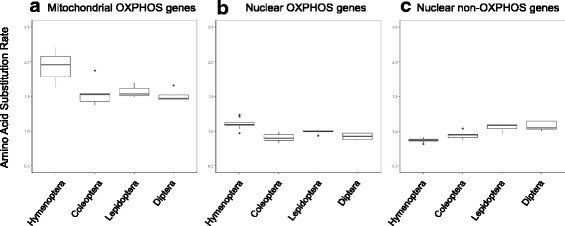
The amino acid substitution rate among insect orders based on concatenated amino acid alignments of (**a**) Mitochondrial OXPHOS genes, (**b**) Nuclear OXPHOS genes, and (**c**) Nuclear non-OXPHOS genes

Using the sum of branch length between two species in the RAxML phylogenetic tree based on the concatenated sequence alignments as approximation for the amino acid substitution rate and using the two-cluster test in LINTREE [40], we found that Hymenoptera exhibited a higher amino acid substitution rate in both mitochondrial genes (Z statistics = 2.20, *p*-value <0.05) and nuclear OXPHOS genes (Z statistics = 7.95, p-value <0.001) than the other three mega-diverse insect orders. In contrast, Hymenoptera had a lower substitution rate in nuclear non-OXPHOS genes (Z statistics = 9.87, p-value <0.001) than the other three insect orders. The general pattern of the concatenated sequences was that the amino acid substitution rate of mitochondrial genes in Hymenoptera (median = 2.12 ± 0.21) was 1.37 times higher than Coleoptera (median = 1.55 ± 0.20), 1.31 times higher than Diptera (median = 1.62 ± 0.04), and 1.27 times higher than Lepidoptera (median = 1.67 ± 0.08) (Fig. 1 and Fig. 2). The amino acid substitution rate of nuclear OXPHOS genes in Hymenoptera (median = 1.09 ± 0.06) was 1.2 times higher than that in Coleoptera (median = 0.90 ± 0.06), 1.17 times higher than Diptera (median = 0.93 ± 0.05), and 1.09 times higher than Lepidoptera (median = 1.00 ± 0.02). On the other hand, the amino acid substitution rate of nuclear non-OXPHOS genes in Hymenoptera (median = 0.87 ± 0.01) was 1.08 times lower compared to Coleoptera (median = 0.94 ± 0.06), 1.2 times lower than Diptera (median = 1.04 ± 0.06) and 1.24 times lower than Lepidoptera (median = 1.08 ± 0.05). On the level of individual genes, 10 out of 13 mitochondrial genes showed a higher amino acid substitution rate in Hymenoptera than the orthologous genes in other insect orders (Table 3). Out of the 23 nuclear OXPHOS genes, 19 evolved faster in Hymenoptera than in the other three insect orders.

**Table 3:** Gene symbol, differences of substitution rate and selection test of each gene

Gene Symbol	Complex	Median Amino acid Substitution Rate differences			PAML Branch Model	PAML Branch-site Model	MEME	TreeSAAP	Sites shared between methods
		Hymenoptera - Coleoptera	Hymenoptera - Lepidoptera	Hymenoptera - Diptera					
mt:ND1	1	−0.37	−0.01	0.14		0	1	0	0
mt:ND2	1	1.91	2.34	2.28		0	2	2	0
mt:ND3	1	−0.13	−0.18	0.25	Lower	0	0	1	0
mt:ND4	1	0.76	0.71	0.81	Higher	0	0	0	0
mt:ND4L	1	1.09	0.59	1.15		0	1	0	0
mt:ND5	1	0.13	0.30	0.33	Lower	0	1	4	0
mt:ND6	1	1.87	3.33	3.81		0	0	0	0
mt:Cyt-b	3	0.60	0.58	0.51		0	1	3	0
mt:CoI	4	0.27	0.31	0.21	Higher	0	1	0	0
mt:CoII	4	0.12	0.22	0.05		0	0	0	0
mt:CoIII	4	−0.60	−0.07	−0.37	Lower	0	0	1	0
mt:ATP6	5	0.16	0.12	0.30		0	2	1	0
mt:ATP8	5	2.38	2.14	2.37		0	0	0	0
ND-42	1	0.97	1.31	1.10	Higher	14	2	14	5
ND-39	1	0.15	0.08	0.31	Higher	11	2	2	2
ND-24	1	0.52	0.24	0.35	Higher	12	0	3	1
ND-75	1	0.14	0.01	0.06		4	1	0	0
ND-B22	1	0.35	0.22	0.74	Higher	0	0	2	0
ND-SGDH	1	0.24	0.07	0.14	Higher	2	1	0	1
ND-B17.2	1	0.33	0.68	0.12	Higher	4	0	0	0
ND-MLRQ	1	0.38	0.78	0.37	Higher	0	1	0	0
ND-23	1	0.12	0.06	0.10	Higher	0	0	0	0
ND-B12	1	−0.13	−0.43	0.00	Higher	0	0	0	0
ND-B16.6	1	0.80	0.55	0.58	Higher	5	1	1	0
ND-B14.5B	1	1.12	2.09	1.33		0	0	0	0
ND-B18	1	0.61	0.17	1.20	Higher	0	0	1	0
ND-PDSW	1	0.48	0.32	0.64	Higher	1	0	0	0
ND-18	1	0.68	0.70	0.66	Higher	0	0	6	0
ND-30	1	0.20	0.06	0.05	Higher	0	0	0	0
UQCR-Q	3	0.19	0.36	0.48	Higher	0	1	1	1
COX5B	4	−0.20	−0.18	−0.08		2	0	0	0
ATPsynF	5	0.45	0.42	0.49	Higher	0	0	0	0
ATPsynγ	5	0.12	0.11	0.30		0	2	0	0
blw	5	−0.04	−0.04	−0.04		0	2	0	0
ATPsynG	5	1.14	0.12	0.90	Higher	0	0	0	0
ATPsynC	5	0.02	−0.02	0.02		0	0	0	0

Gene symbol is the name of the gene in *Drosophila melanogaster.* Complex indicates the location of the gene product in the OXPHOS complex. The median of amino acid substitution rate differences is the differences between the median substitution rate of Hymenoptera compared to the other three orders. Positive or negative values indicate the amino acid substitution rate of a gene is higher or lower in Hymenoptera than in the other insect orders. For PAML branch model, “Higher” or “Lower” means the gene has a significant higher or lower *dN/dS* ratio in Hymenoptera than in the other three orders based on PAML branch model test. For PAML branch-site model and MEME, the number indicates the number of codons under positive selection with *dN/dS* > 1. For TreeSAAP, the number indicates the number of amino acids under positive selection. The last column indicates the number of codon sites or amino acids found by at least two methods in PAML branch-site model, MEME, or TreeSAAP

We also tested the differences in evolutionary rates among concatenated mitochondrial, nuclear OXPHOS and nuclear non-OXPHOS genes in the same insect order based on the terminal branches with the Wilcoxon rank sum test. In Hymenoptera, mitochondrial OXPHOS genes had a higher amino acid substitution rate than nuclear OXPHOS genes (*p*-value = 0.012) and nuclear non-OXPHOS genes (p-value = 0.012); nuclear non-OXPHOS genes had a lower substitution rate than mitochondrial OXPHOS genes (p-value = 0.012) and than nuclear OXPHOS genes (p-value = 0.012). In Diptera, nuclear non-OXPHOS genes had a higher amino acid substitution rate than nuclear OXPHOS genes (*p*-value = 0.047). Comparisons between gene groups in other orders were not significant.

### Tests for the signature of positive selection on OXPHOS genes

Among the 13 mitochondrial genes analyzed, only 2 had a higher *dN*/*dS* ratio in Hymenoptera than in the three other orders based on PAML branch test (Table 3). PAML branch-site model, MEME, and TreeSAAP revealed 0, 9 and 12 sites under positive selection, respectively. None of these sites were detected by more than one method.

Based on the results of the PAML branch test, 17 out of 23 nuclear OXPHOS genes had elevated *dN*/*dS* ratios in Hymenoptera compared to the other three insect orders (Table 3, Additional file 4: Table S3). For the site-specific positive selection test, 17 nuclear OXPHOS genes revealed a total of 108 codon sites under positive selection (Additional file 5: Table S4). A total of 55, 22, and 42 codon sites (out of 4127 codon sites) were found to be under positive selection by the PAML branch-site model, by MEME, and by TreeSAAP, respectively. Ten codon sites were found under positive selection by at least two methods (Table 3, Additional file 5: Table S4). Among the 1413 nuclear non-OXPHOS genes, 516 genes were found with positive selection with PAML branch-site model. A total of 2823 codon sites (out of 343,228 codon sites) were found under positive selection with PAML branch-site model.

## Discussion

Interactions between mitochondrial and nuclear genes have been implicated in a number of important evolutionary processes, for example, establishing reproductive isolation between species by giving rise to genic incompatibilities and driving the evolution of sex [21, 27, 49] . A common assertion is that these interactions should couple the patterns of molecular evolution between mitochondrial and nuclear OXPHOS genes. However, it is not clear what mechanisms are driving the observed elevated substitution rate in nuclear OXPHOS genes. One leading hypothesis is that these elevated rates may be a response to selection from rapidly evolving mitochondrial genes [19, 20]. To test this hypothesis, we compared the evolutionary rate of nuclear OXPHOS and non-OXPHOS genes in Hymenoptera, a lineage with rapidly evolving mitochondrial genes, to the genes in three other holometabolous insect orders with more slowly evolving mitochondrial genes (i.e. Coleoptera, Diptera, and Lepidoptera). We leverage the rapid rate of molecular evolution in the mitochondrial genome of Hymenoptera to test for evidence of positive selection on the nuclear OXPHOS genes.

### Hymenoptera exhibited a high amino acid substitution rate in their nuclear OXPHOS genes

By comparing the amino acid sequences of OXPHOS genes of Hymenoptera to those of other holometabolous insects, we found that Hymenopterans exhibit a significantly elevated amino acid substitution rate in their mitochondrial and nuclear OXPHOS genes, but not in their nuclear non-OXHPOS genes (Fig. 2). Our finding is consistent with a previous study on Hymenoptera [26], which noted an elevated amino acid substitution rate in mitochondrial OXPHOS genes, but not in four nuclear non-OXPHOS genes. By increasing the number of nuclear non-OXPHOS genes to 1413, we demonstrated that the lower rate of substitution in non-OXPHOS genes was not due to a sampling artifact in the previous study [26]. In this much larger set of genes, we found the amino acid substitution patterns in nuclear non-OXPHOS genes showed a lower substitution rate in Hymenoptera compared to other insect orders. In addition, we found that 19 of the representative 23 nuclear OXPHOS genes show an elevated amino acid substitution rate. This higher amino acid substitution rate in nuclear OXPHOS genes is likely representative for Hymenoptera as the nine hymenopterans have a comprehensive phylogenetic coverage of the order (see Peters et al. [50] for more information on their phylogenetic position within Hymenoptera).

The evolution of nuclear OXPHOS genes in Hymenoptera shows a different pattern from the three other insect orders, where nuclear OXPHOS genes evolve faster than non-OXPHOS genes in Hymenoptera, but slower than non-OXPHOS genes (Diptera) or similar in substitution rates (Coleoptera and Lepidoptera). In the nuclear genome, the evolution of OXPHOS genes could be under both functional constraints and selection pressure due to changes in the mitochondrial genome [6, 51]. If the selection pressure due to changes in the mitochondrial genome is weak, as seemingly in Diptera, Coleoptera and Lepidoptera, where the evolutionary rates of mitochondrial genes are slower than that in Hymenoptera, functional constraints would play a major role. In contrast, if selection pressure due to rapid changes in the mitochondrial genome is strong, selection would play a more important role than functional constraints.

### Evidence of positive selection on nuclear OXPHOS genes

Since we found evidence of elevated rates of substitution in nuclear OXPHOS genes of Hymenoptera, we used a series of four tests to detect the signature of positive selection in these genes. Applying the PAML branch model test, we found that 17 out of 23 nuclear OXPHOS genes had a higher *dN*/*dS* ratio in Hymenoptera than the orthologous genes from the other insect orders (Table 3, Additional file 4: Table S3). In principle, a high *dN*/*dS* ratio could be caused by either positive selection or relaxed functional constraints and it is difficult to distinguish between these two possibilities by looking exclusively at the *dN*/*dS* ratio. Both positive selection and relaxed functional constraints can lead to the fixation of non-synonymous mutations in a population [6, 19, 27]. Positive selection is more likely to lead to the fixation of beneficial non-synonymous mutations, whereas relaxed functional constraints are expected to decrease the degree of purifying selection, which can lead to the fixation of deleterious mutations. Given the importance of the OXPHOS system for aerobic organisms, nuclear OXPHOS genes are less likely under relaxed functional constraints [27, 52].

We used the PAML branch-site test, MEME and TreeSAAP to identify codon or amino acid sites that have been under positive selection. These approaches are based on different models. The branch-site model of PAML tests for codon sites under positive selection in specific branches with *a prior* assumption of which branches are under selection [43]. The MEME test is based on a mixed-effect model, which tests for codon sites under positive selection in all branches without an *a prior* expectation [44]. TreeSAAP tests for amino acid sites under positive selection based on the structural and biochemical properties of amino acids [47]. The branch-site model of PAML found the highest number of codon or amino acid sites under positive selection. MEME found the lowest number of sites under positive selection. Although these three approaches are based on different models, ten sites were detected as under positive selection by at least two approaches. The proportion of codon sites under positive selection in Hymenoptera detected with PAML branch-site model is significantly higher (*p*-value <0.001 with Fisher’s exact test) in nuclear OXPHOS genes (1.33%) than in nuclear non-OXPHOS genes (0.82%). Overall, we found evidence of positive selection at a few specific amino acid positions in nuclear OXPHOS genes (details about sites under positive selection can be found in Additional file 5: Table S4). The three genes (ND-39, ND24, and ND-42 gene) with the highest number of sites under positive selection are known to have functional importance. ND-39 is relevant to mitochondrial-nuclear incompatibility among *Nasonia* species [27]. Another interacting gene, ND-24, is a core subunit of the OXPHOS complex I, which binds and oxidizes NADH [53]. In the ND-42 gene, a deleterious mutation at a single amino acid site (Gln142Arg) is known to disturb complex I assembly, potentially causing Leigh disease [54]. We found that an amino acid site immediately adjacent to the deleterious locus linked to Leigh disease shows evidence of positive selection in Hymenoptera (Additional file 5: Table S4; Codon 106 in the codon alignment).

Although there is evidence of positive selection based on the elevated *dN*/*dS* ratio and codon sites under positive selection, the positive selection pattern is not consistent with the observed elevated amino acid substitution rate. In our results, there are nuclear OXPHOS genes with a high amino acid substitution rate that do not show a clear signature of positive selection, such as the ATPsynγ gene and the ND-B14.5B gene. For example, the substitution rate of the ND-B14.5B gene in Hymenoptera is 1.4–2.2 times higher than the rate in other insect orders, while the *dN*/*dS* ratio of this gene in Hymenoptera is not significantly higher than that in other insect orders. No specific sites in the ND-B14.5B gene were found to be under positive selection in Hymenoptera.

The inconsistent pattern between the amino acid substitution rate and the *dN*/*dS* ratio could be the consequence of failure to detect positive selection. The divergence time between Hymenoptera and the other three insect orders is between 345 and 355 million years [28, 55]. With this deep divergence time, nucleotide substitutions are likely highly saturated [56], making it difficult to detect positive selection. In addition, OXPHOS genes are under strong purifying selection [27], and the signal of episodic positive selection is difficult to detect in a background of strong purifying selection. A long divergence time and presence of strong purifying selection make it difficult to conclude whether the observed high amino acid substitution rate is indeed caused by positive selection. Future studies on populations with short divergence times could help detect positive selection signals (if there are any) and to test the role of positive selection in the evolution of the OXPHOS in Hymenoptera, for example, when studying populations of the same species that differ in the substitution rate of their mitochondrial genes [20].

Our study sheds light on the idea that the high amino acid substitution rate in nuclear OXPHOS genes was driven by positive selection in Hymenoptera. In fact, we found that a small number of sites in nuclear OXPHOS genes of Hymenoptera have evolved under positive selection (Table 3). The source of positive selection could be the high rate of amino acid substitution in the mitochondrial genome [27]. Based on our finding that only nuclear OXPHOS genes (and not nuclear non-OXPHOS genes) have elevated amino acid substitution rates, it is less likely that small effective population sizes in Hymenoptera, resulting from a parasitoid life style [23] and/or haplodiploidy [25], have caused the high amino acid substitution rate in nuclear OXPHOS genes due to drift, as both factors would have genome-wide effect on substitution rates.

## Conclusions

In this study, we used published transcriptome sequence data to provide insights into the evolution of OXPHOS genes in the four mega-diverse insect orders Coleoptera, Diptera, Hymenoptera, and Lepidoptera. We found that mitochondrial and nuclear OXPHOS genes of Hymenoptera exhibited significant higher average substitution rates than the genes of other insect orders. The higher substitution rate in nuclear OXPHOS genes of Hymenoptera could be, at least in part, explained by positive selection. However, positive selection alone cannot explain the elevated rate of molecular evolution of nuclear OXPHOS genes in Hymenoptera.

## Additional files

Additional file 1: Table S1. The list of OXPHOS gene Symbols and the corresponding OrthoDB Gene ID. (XLSX 9 kb)

Additional file 2: Table S2. Genes that are missing in transcriptome data set. (XLSX 11 kb)

Additional file 3: Data, script and example files used in the study. (DOCX 97 kb)

Additional file 4: Table S3. dN/dS ratio estimates from PAML branch model. (XLSX 10 kb)

Additional file 5: Table S4. Codon sites that were detected under positive selection in OXPHOS genes based on the PAML branch-site model, MEME and TreeSAAP. (XLSX 11 kb)

## Abbreviations

*dN*/*dS*: The ratio of the number of non-synonymous nucleotide substitutions per non-synonymous site to the number of synonymous nucleotide substitutions per synonymous site
OXPHOS: Oxidative phosphorylation

## References

[1] Schmidt TR, Wu W, Goodman M, Grossman LI (2001). Evolution of nuclear-and mitochondrial-encoded subunit interaction in cytochrome c oxidase. Mol Biol Evol.

[2] Rand DM, Haney RA, Fry AJ (2004). Cytonuclear coevolution: the genomics of cooperation. Trends Ecol Evol.

[3] Bar-Yaacov D, Blumberg A, Mishmar D (1819). Mitochondrial-nuclear co-evolution and its effects on OXPHOS activity and regulation. Biochim Biophys Acta BBA-Gene Regul Mech.

[4] Hatefi Y (1985). The mitochondrial electron transport and oxidative phosphorylation system. Annu Rev Biochem.

[5] Zheng J (2012). Energy metabolism of cancer: glycolysis versus oxidative phosphorylation. Oncol Lett.

[6] Zhang F, Broughton RE (2013). Mitochondrial–nuclear interactions: compensatory evolution or variable functional constraint among vertebrate oxidative phosphorylation genes?. Genome Biol. Evol..

[7] Tripoli G, D’Elia D, Barsanti P, Caggese C (2005). Comparison of the oxidative phosphorylation (OXPHOS) nuclear genes in the genomes of Drosophila Melanogaster, Drosophila pseudoobscura and Anopheles gambiae. Genome Biol.

[8] Porcelli D, Barsanti P, Pesole G, Caggese C (2007). The nuclear OXPHOS genes in insecta: a common evolutionary origin, a common cis-regulatory motif, a common destiny for gene duplicates. BMC Evol Biol.

[9] Gibson J, Niehuis O, Peirson B, Cash E, Gadau J (2013). Genetic and developmental basis of F2 hybrid breakdown in Nasonia parasitoid wasps. Evolution.

[10] Ellison C, Niehuis O, Gadau J (2008). Hybrid breakdown and mitochondrial dysfunction in hybrids of Nasonia parasitoid wasps. J Evol Biol.

[11] Barreto FS, Burton RS (2013). Elevated oxidative damage is correlated with reduced fitness in interpopulation hybrids of a marine copepod. Proc R Soc Lond B Biol Sci.

[12] Brown WM, George M, Wilson AC (1979). Rapid evolution of animal mitochondrial DNA. Proc Natl Acad Sci.

[13] Vawter L, Brown WM (1986). Nuclear and mitochondrial DNA comparisons reveal extreme rate variation in the molecular clock. Science.

[14] Lynch M (1996). Mutation accumulation in transfer RNAs: molecular evidence for Muller’s ratchet in mitochondrial genomes. Mol Biol Evol.

[15] Lynch M, Blanchard JL (1998). Deleterious mutation accumulation in organelle genomes. Genetica.

[16] Barr CM, Neiman M, Taylor DR (2005). Inheritance and recombination of mitochondrial genomes in plants, fungi and animals. New Phytol.

[17] Neiman M, Taylor DR. The causes of mutation accumulation in mitochondrial genomes. Proc. R. Soc. Lond. B Biol. Sci. 2009;rspb–2008. 10.1098/rspb.2008.1758 PMC2660971 19203921

[18] Lynch M, Gabriel W. Mutation load and the survival of small populations. Evolution. 1990:1725–37. 10.1111/j.1558-5646.1990.tb05244.x 28567811

[19] Havird JC, Whitehill NS, Snow CD, Sloan DB (2015). Conservative and compensatory evolution in oxidative phosphorylation complexes of angiosperms with highly divergent rates of mitochondrial genome evolution. Evolution.

[20] Havird JC, Trapp P, Miller C, Bazos I, Sloan DB. Causes and consequences of rapidly evolving mtDNA in a plant lineage. Genome Biol Evol. 2017; 10.1093/gbe/evx010 PMC5381668 28164243

[21] Hill GE (2015). Mitonuclear ecology. Mol Biol Evol.

[22] Sloan DB, Triant DA, Wu M, Taylor DR (2013). Cytonuclear interactions and relaxed selection accelerate sequence evolution in organelle ribosomes. Mol Biol Evol.

[23] Castro L, Austin A, Dowton M (2002). Contrasting rates of mitochondrial molecular evolution in parasitic Diptera and hymenoptera. Mol Biol Evol.

[24] Talavera G, Vila R (2011). What is the phylogenetic signal limit from mitogenomes? The reconciliation between mitochondrial and nuclear data in the Insecta class phylogeny. BMC Evol Biol.

[25] Cameron SL (2014). Insect mitochondrial genomics: implications for evolution and phylogeny. Annu Rev Entomol.

[26] Kaltenpoth M, Corneli PS, Dunn DM, Weiss RB, Strohm E, Seger J (2012). Accelerated evolution of mitochondrial but not nuclear genomes of hymenoptera: new evidence from crabronid wasps. PLoS One.

[27] Gibson JD, Niehuis O, Verrelli BC, Gadau J (2010). Contrasting patterns of selective constraints in nuclear-encoded genes of the oxidative phosphorylation pathway in holometabolous insects and their possible role in hybrid breakdown in Nasonia. Heredity.

[28] Misof B, Liu S, Meusemann K, Peters RS, Donath A, Mayer C (2014). Phylogenomics resolves the timing and pattern of insect evolution. Science.

[29] Waterhouse RM, Zdobnov EM, Tegenfeldt F, Li J, Kriventseva EV (2011). OrthoDB: the hierarchical catalog of eukaryotic orthologs in 2011. Nucleic Acids Res.

[30] Zhou X, Li Y, Liu S, Yang Q, Su X, Zhou L (2013). Ultra-deep sequencing enables high-fidelity recovery of biodiversity for bulk arthropod samples without PCR amplification. Gigascience.

[31] Nygaard S, Zhang G, Schiøtt M, Li C, Wurm Y, Hu H (2011). The genome of the leaf-cutting ant Acromyrmex Echinatior suggests key adaptations to advanced social life and fungus farming. Genome Res.

[32] Elsik CG, Tayal A, Diesh CM, Unni DR, Emery ML, Nguyen HN (2015). Hymenoptera genome database: integrating genome annotations in HymenopteraMine. Nucleic Acids Res.

[33] Bonasio R, Zhang G, Ye C, Mutti NS, Fang X, Qin N (2010). Genomic comparison of the ants Camponotus Floridanus and Harpegnathos Saltator. Science.

[34] Wei S, Shi M, Sharkey MJ, van Achterberg C, Chen X (2010). Comparative mitogenomics of Braconidae (Insecta: hymenoptera) and the phylogenetic utility of mitochondrial genomes with special reference to Holometabolous insects. BMC Genomics.

[35] Edgar RCMUSCLE (2004). Multiple sequence alignment with high accuracy and high throughput. Nucleic Acids Res.

[36] Castresana J (2000). Selection of conserved blocks from multiple alignments for their use in phylogenetic analysis. Mol Biol Evol.

[37] Stamatakis A (2014). RAxML version 8: a tool for phylogenetic analysis and post-analysis of large phylogenies. Bioinformatics.

[38] R Core Team. R: A Language and Environment for Statistical Computing [Internet]. Vienna, Austria: R Foundation for Statistical Computing; 2016. Available from: https://www.R-project.org/.

[39] Yu G, Smith D, Zhu H, Guan Y, Lam TT-Y. ggtree: an R package for visualization and annotation of phylogenetic trees with their covariates and other associated data. Methods Ecol Evol 2017;8:28–36.

[40] Takezaki N, Rzhetsky A, Nei M (1995). Phylogenetic test of the molecular clock and linearized trees. Mol Biol Evol.

[41] Holm SA. Simple sequentially rejective multiple test procedure. Scand J Stat. 1979:65–70.

[42] Yang ZPAML (2007). 4: phylogenetic analysis by maximum likelihood. Mol Biol Evol.

[43] Zhang J, Nielsen R, Yang Z (2005). Evaluation of an improved branch-site likelihood method for detecting positive selection at the molecular level. Mol Biol Evol.

[44] Murrell B, Wertheim JO, Moola S, Weighill T, Scheffler K, Pond SLK (2012). Detecting individual sites subject to episodic diversifying selection. PLoS Genet.

[45] Pond SLK, Muse SV. HyPhy: hypothesis testing using phylogenies. Stat. Methods Mol. Evol. Spring; 2005. p. 125–181.

[46] Lu A, Guindon S (2013). Performance of standard and stochastic branch-site models for detecting positive selection amongst coding sequences. Mol Biol Evol.

[47] Woolley S, Johnson J, Smith MJ, Crandall KA, McClellan DA (2003). TreeSAAP: selection on amino acid properties using phylogenetic trees. Bioinformatics.

[48] Maldonado E, Sunagar K, Almeida D, Vasconcelos V, Antunes A. IMPACT_S: integrated multiprogram platform to analyze and combine tests of selection. PLoS One 2014;9:e96243. 10.1371/journal.pone.0096243 PMC4203653 25329307

[49] Burton RS, Barreto FSA (2012). Disproportionate role for mtDNA in Dobzhansky–Muller incompatibilities?. Mol Ecol.

[50] Peters RS, Krogmann L, Mayer C, Donath A, Gunkel S, Meusemann K, et al. Evolutionary history of the hymenoptera. Curr Biol. 2017; 10.1016/j.cub.2017.01.027 28343967

[51] De Grassi A, Lanave C, Saccone C (2008). Genome duplication and gene-family evolution: the case of three OXPHOS gene families. Gene.

[52] Mitterboeck TF, Liu S, Adamowicz SJ, Fu J, Zhang R, Song W, et al. Positive and relaxed selection associated with flight evolution and loss in insect transcriptomes. Gigascience. 2017; 10.1093/gigascience/gix073 PMC5632299 29020740

[53] Mimaki M, Wang X, McKenzie M, Thorburn DR, Ryan MT (1817). Understanding mitochondrial complex I assembly in health and disease. Biochim Biophys Acta BBA-Bioenerg.

[54] Hoefs SJ, van Spronsen FJ, Lenssen EW, Nijtmans LG, Rodenburg RJ, Smeitink JA (2011). NDUFA10 mutations cause complex I deficiency in a patient with Leigh disease. Eur J Hum Genet.

[55] Wiegmann BM, Trautwein MD, Kim J-W, Cassel BK, Bertone MA, Winterton SL (2009). Single-copy nuclear genes resolve the phylogeny of the holometabolous insects. BMC Biol.

[56] Gharib WH, Robinson-Rechavi M (2013). The branch-site test of positive selection is surprisingly robust but lacks power under synonymous substitution saturation and variation in GC. Mol Biol Evol.

