# Supplementary material for: The molecular evolutionary dynamics of oxidative phosphorylation (OXPHOS) genes in Hymenoptera
Source: BMC Evol Biol. 2017 Dec 28;17:269. doi: 10.1186/s12862-017-1111-z (PMC5745899; doi:10.1186/s12862-017-1111-z)
Supplement: Supplementary file 3 — Data, script and example files used in the study. (DOCX 97 kb) [file 12862_2017_1111_MOESM3_ESM.docx]

**The Molecular Evolutionary Dynamics of Oxidative Phosphorylation (OXPHOS) Genes in Hymenoptera**

1. **Data repository**

figshare (https://doi.org/10.6084/m9.figshare.5378071)

1. **Perl scripts used in the analysis**

Codon alignment:

<https://github.com/lyy005/codon_alignment/>

Translate cds to amino acid sequences:

<https://github.com/lyy005/cds2aa>

1. **Packages used for the analysis**

MUSCLE:

<http://www.drive5.com/muscle/>

LINTREE package:

<http://www.personal.psu.edu/nxm2/software.htm>

TreeSAAP:

<http://dna.cs.byu.edu/treesaap/>

1. **Command for analysis**

###

# Sequence alignment for Nuclear genes

###

perl codon_alignment.pl Example.NonStopCodon.fas 1 0

###

# Sequence alignment for Mitochondrial genes

###

perl codon_alignment.pl Example.NonStopCodon.fas 5 0

###

# Gblock:

###

Gblocks Example.NonStopCodon.fas.nt.cleanup.aln -t=c -b4=5 -b5=a -e=-gb1

less -S Example.NonStopCodon.fas.nt.cleanup.aln-gb1| perl -e '$/ = ">"; <>; while(<>){chomp; @line=split/\n+/; my $name = shift @line; @name=split/\s+/, $name; my $seq = join "",@line; $seq =~s/\s+//g; print ">$name[0]\n$seq\n";}' > Example.NonStopCodon.nt.GBlocks.fas

###

# CDS to amino acid sequences

###

perl cds2aa.pl Example.NonStopCodon.nt.GBlocks.fas NUC Example.NonStopCodon.aa.GBlocks.fas

###

# Tree building:

###

raxmlHPC-PTHREADS -f a -m PROTGAMMAAUTO -p 12345 -x 12345 -\# 100 -s Example.NonStopCodon.aa.GBlocks.fas -n mito -o ACYRTHOS -T 8

###

# Estimating tip to root branch length:

###

Data and examples in OXPHOS_data_submission/branch_length.R

###

# LINTREE test:

###

Data and examples in OXPHOS_data_submission/LINTREE folder

tpcv MT.Concatenated.Gblock.aa.rm3spp.phylip.fas.reorder -tMT.all.lintree -d3 -o 29

tpcv NUC.Concatenated.Gblock.aa.phylip.fas.reorder -tNUC.all.lintree -d3 -o 32

tpcv Control.Concatenated.Gblock.aa.phylip.fas.reorder -tControl.all.lintree -d3 -o 32

###

# PAML branch model:

###

Data and examples in OXPHOS_data_submission/PAML_branch folder

###

# PAML branch-site model

###

Data and examples in OXPHOS_data_submission/PAML_branch-site folder

###

# MEME

###

Data and examples in OXPHOS_data_submission/MEME folder
